# An Overview of the Strategies to Boost SARS-CoV-2-Specific Immunity in People with Inborn Errors of Immunity

**DOI:** 10.3390/vaccines12060675

**Published:** 2024-06-18

**Authors:** Emma Chang-Rabley, Menno C. van Zelm, Emily E. Ricotta, Emily S. J. Edwards

**Affiliations:** 1 The Laboratory of Clinical Immunology and Microbiology, National Institute of Allergy and Infectious Diseases, National Institutes of Health, Bethesda, MD 20892, USA; 2 Allergy and Clinical Immunology Laboratory, Department of Immunology, Central Clinical School, Monash University, Melbourne, VIC 3800, Australia; 3 The Jeffrey Modell Diagnostic and Research Centre for Primary Immunodeficiencies in Melbourne, Melbourne, VIC 3000, Australia; 4 Department of Immunology, Erasmus MC, University Medical Center, 3015 GD Rotterdam, The Netherlands; 5 Department of Preventive Medicine and Biostatistics, Uniform Services University of the Health Sciences, Bethesda, MD 20814, USA

**Keywords:** COVID-19 vaccination, SARS-CoV-2-specific antibody therapies, immune memory, inborn errors of immunity, breakthrough infections

## Abstract

The SARS-CoV-2 pandemic has heightened concerns about immunological protection, especially for individuals with inborn errors of immunity (IEI). While COVID-19 vaccines elicit strong immune responses in healthy individuals, their effectiveness in IEI patients remains unclear, particularly against new viral variants and vaccine formulations. This uncertainty has led to anxiety, prolonged self-isolation, and repeated vaccinations with uncertain benefits among IEI patients. Despite some level of immune response from vaccination, the definition of protective immunity in IEI individuals is still unknown. Given their susceptibility to severe COVID-19, strategies such as immunoglobulin replacement therapy (IgRT) and monoclonal antibodies have been employed to provide passive immunity, and protection against both current and emerging variants. This review examines the efficacy of COVID-19 vaccines and antibody-based therapies in IEI patients, their capacity to recognize viral variants, and the necessary advances required for the ongoing protection of people with IEIs.

## 1. Introduction

Since its emergence in November 2019, severe acute respiratory coronavirus-2 (SARS-CoV-2) has caused over 775 million confirmed cases of coronavirus disease 2019 (COVID-19) and over 7 million deaths worldwide [1]. The global fatality rates range across countries and age groups, from <1% in those aged <50 years to ~4–25% in healthy individuals 60–80 years of age [2,3].

SARS-CoV-2 is an enveloped virus with a linear RNA genome belonging to the *Coronaviridae* family [4]. The infectious nature of SARS-CoV-2 potentiated the quick establishment of a pandemic, rather than the epidemic (SARS-CoV, MERS-CoV) or endemic (seasonal coronaviruses) outbreaks of its predecessors [5,6].

Infection is established when the spike receptor binding domain (RBD) of the SARS-CoV-2 virus engages the angiotensin-converting enzyme 2 (ACE2) receptor on the surface of host T-cells, especially lung, heart, and kidney cells [7,8]. While, in most healthy individuals, SARS-CoV-2 infection causes asymptomatic or mild respiratory disease including fever, cough, and lethargy, a subset of individuals mount an inappropriately strong inflammatory response to high viral loads, resulting in severe illness, hospitalization, respiratory support, and in some cases death [9,10]. This leads to overwhelming concern that people with defective immunity, particularly those with rare inherited immunodeficiencies (approximately 1 in 10,000 people) [11] or more common acquired immunodeficiencies (approximately 1 in 1200 people) [12], may be at increased risk of severe COVID-19 disease.

Whilst public health measures, such as mask wearing, hand washing, social distancing, and shielding, have undoubtedly helped to protect people with inborn errors of immunity (IEIs), this has posed other issues, including feelings of isolation and adverse impacts on quality of life [13,14]. As such, the medical and scientific community rapidly pivoted to combat the global pandemic by providing novel and innovative solutions for SARS-CoV-2 testing, vaccination, and therapies to treat disease and help cover the gap in immunity in an effort to reduce virus transmission and COVID-19 disease severity. Here, we provide an in-depth overview of the current understanding of COVID-19 vaccine efficacy in people with IEI, the efficacy of SARS-CoV-2-specific antibody products, and new insights and technological advances that can enhance protection for people with IEI or other immunocompromised conditions in the changing SARS-CoV-2 landscape [15].

### 1.1. Inborn Errors of Immunity (IEI)

At present, genetic variants in more than 485 genes are known to cause IEIs. Patients with IEIs can be broadly classified into nine groups capturing the diversity of clinical, genetic, and immunological phenotypes of disease, plus an additional tenth category covering IEI phenocopies. Groups are defined by the nature of the rare genetic defect identified, the immune cell(s) affected, and infections to which individuals exhibit increased susceptibility [16]. Of these nine categories, the most prevalent is predominantly antibody deficiency (PAD) [17,18], including patients with Common Variable Immunodeficiency (CVID) and agammaglobulinemia. Other categories include combined immunodeficiencies (CID) and diseases of immune dysregulation known to have defects in multiple immune cell lineages. A common presenting feature in PAD is profound antibody deficiency, poor responses to vaccination, and increased susceptibility to sinopulmonary infections underpinned by defects in B-cell development, differentiation, and function [17,19,20]. It is generally accepted that many of these diseases are accompanied by defects in other immune cells involved in innate and adaptive responses. These include T-cells, which are integral for robust generation of imunity to infectious agents and may have a key role in vaccine efficacy for some pathogens, particularly those of viral origin. As a result, in people with PAD and other IEIs, reduced vaccine efficacy can result in increased susceptibility to vaccine-preventable disease. For these individuals, preventative therapy centers around tackling antibody deficiency, including lifelong immunoglobulin replacement therapy (IgRT), annual vaccinations (e.g., for influenza), and prophylactic antibiotics, in a complicated attempt to reduce the infectious burden [21,22]. Thus, the emergence of the novel SARS-CoV-2 virus presents unprecedented challenges for protecting and treating those with IEIs [17,20].

### 1.2. COVID-19 Infection in People with IEI

While, in healthy individuals, SARS-CoV-2 infection generates functional memory B- (Bmem), memory CD4^+^ T- (CD4^+^ Tmem), and CD8^+^ T-cell (CD8^+^ Tmem) responses, as well as neutralizing antibodies [5,6,23], some of these responses are perturbed in people with IEI, resulting in more severe disease outcomes [3]. These have been extensively reviewed elsewhere and will not be described in depth here. Briefly, Tangye et al. recently reported that ~1330 infections with SARS-CoV-2 in IEI had been published to date, with 60% of reported individuals diagnosed with antibody deficiency [3]. Among these, a case fatality rate of 8.5% was documented in people with IEIs as compared to ~1.1% in the general population. It was noted that the case fatality rate for children 0–19 years old with IEIs was up to 100 times higher than that observed for those of the same age in the general population [3,24].

Within the IEI population, individuals with defects in genes including *AIRE* (autoimmune regulator), *IRF7* (interferon regulatory factor 7), and *IFNAR1/2* (interferon-α/β receptor) had significantly higher COVID-19 case fatality rates, clearly demonstrating that genetics can adversely impact patient outcomes to SARS-CoV-2 infection [3]. In particular, individuals with biallelic variants in *AIRE* experienced the most severe COVID-19 disease as a consequence of the production of neutralizing antibodies to cytokines, including type I interferon (IFN). These anti-IFN antibodies prevent binding of IFN-α or β to its receptor (IFNAR), thereby inhibiting the pivotal role of IFNs in mediating a host response to viruses [25]. Furthermore, SARS-CoV-2 has unmasked undiagnosed IEI in otherwise asymptomatic individuals, with these individuals presenting with life-threatening COVID-19. The first identified genetic cause of severe COVID-19 disease was published in 2020 by van der Made et al. Here, rare X-linked (XL) loss-of-function (LOF) variants in the gene encoding Toll-like receptor 7 (*TLR7*) were identified in four young males with critical COVID-19 disease [26]. These variants were shown to cause impaired type I and II IFN responses, known to be crucial for immune responses against viruses. Later studies have additionally revealed novel, rare XL LOF variants in *TLR7* in young males critically ill with COVID-19, with these defects being shown to underlie severe COVID-19 disease in 1–2% of men under 60 years of age [26,27,28,29]. Additionally, *TLR7* genetic variants in the *TLR3* gene have been found to compromise local type I IFN-mediated innate immune responses. Together, variants in these genes are estimated to be the cause of severe COVID-19 in up to 5% of people under 70 years of age [26,27,30].

Additional studies have identified additional loci where enrichment of rare autosomal recessive (AR) or autosomal dominant (AD) variants in *IFNAR1* (AR/AD)*, IFNAR2* (AD), *IRF3* (AD), *IRF7* (AR/AD), *TBK1* (AD), *TICAM1* (AD), *TLR3* (AD), and *UNC931* (AD), predispose ~3.5% of individuals with severe SARS-CoV-2 infection to critical COVID-19 pneumonia [31]. These genes are known to control TLR3 and IRF7-dependent induction and enhancement of type I IFN signaling, showing the important role of these pathways in the control of SARS-CoV-2 infection. Thus, this suggests that type I IFN administration might reduce disease severity if administered early in the course of the disease [31]. These findings were subsequently followed by the discovery of additional rare variants in *IFNAR1* (AR) [32,33], *TNFRSF13B* (AD), and *TBK1* (AD) underlying severe COVID-19 [34].

Strikingly, although only a few individuals have been identified with rare AR *IFNAR1* or *IFNAR2* variants, COVID-19 lethality rates are 57% in these individuals [31,32,33,35]. *IFNAR1* and *IFNAR2* encode the α and β chain of the type I interferon (IFN)-α/β receptor (IFNAR), respectively, which, along with IFN-I, form the IFNAR receptor. Upon binding of IFN-α or β to the IFNAR receptor, the IFNAR1 chain binds tyrosine kinase-2 (TYK2) and IFNAR2 interacts with Janus Kinase-1 (JAK1) and signal transduction and transcription activation (STAT), mediating downstream JAK/STAT (primarily STAT1 and STAT2) signal transduction and resulting in the IFN-I induced cellular response [36].

Further, an international study has identified recessive defects, including XL *TLR7* and AR *IFNAR1*, *STAT2,* and *TYK2*, in 10% of pediatric patients hospitalized with COVID-19 pneumonia [37]. Together, the predisposition of individuals with variants in the aforementioned genes to severe COVID-19 disease demonstrates the critical role of type I IFN in host defense against SARS-CoV-2, whilst this pathway is redundant for other infectious agents. Interestingly, a more recent retrospective cohort study by Ngyuen et al. found that many studies of infection outcomes in IEI did not control for factors classified as social determinants of health, such as race/ethnicity [38]. In their cohort of various combined, humoral, or innate immunodeficiencies and ages (2 months to 69 years), they identified an association between an increased risk of hospitalization and race, ethnicity, obesity, and neurological disease. The authors posit that these findings have important implications for current treatment and COVID-19 disease management guidelines for IEI populations, which center around cellular and genetic mechanisms of severe disease risk.

### 1.3. SARS-CoV-2 Variants

Over the pandemic, multiple SARS-CoV-2 variants have evolved, with the first, Alpha, detected in September 2020 (Table S1). Viral variants contain mutations that are advantageous for viral spread, e.g., increased infectivity, viral fitness, or escape from immune responses [39]. Multiple predisposing factors, including geographical location, environmental and economic conditions, and access to vaccination, favor viral diversification, thus leading to the emergence of variants in the general population [40]. Indeed, this concern extends to the unvaccinated, and inequitable global vaccine access may play a role in the ongoing development of SARS-CoV-2 variants. There is also evidence that the delayed viral clearance observed in people with immune disorders contributes to the emergence of viral variants [41,42,43,44].

Whilst over 30 different SARS-CoV-2 genetic clades have been identified to date, not all have been cause for heightened concern or attention. Therefore, the World Health Organization (WHO) monitored variants with increased viral transmissibility or virulence or escape from the host antibody. For example, the Omicron BA.1 variant, which emerged in November 2021, has 37 spike protein mutations, resulting in increased ACE2 binding affinity and decreased humoral immune recognition [45]. To date, the Omicron lineage is the most divergent variant due to the high number of mutations it harbors, with all sublineages maintaining properties of increased immune evasion and preference for binding to ACE2 compared to earlier SARS-CoV-2 variants [46,47] (Table S1).

**Figure 1:**
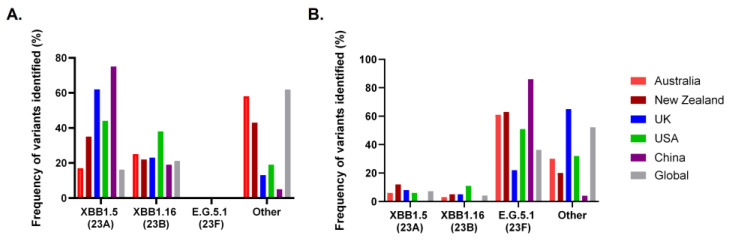
Shifting predominance of SARS-CoV-2 variants over time and in different geographical locations worldwide. SARS-CoV-2 variant predominance in different countries and globally (**A**) as at 10 July 2023 and (**B**) 18 December 2023. Global data from [49] and individual country data from [50].

Due to the ongoing evolution of SARS-CoV-2 variants, there was a shift in variant predominance from Omicron XBB.1.5 and XBB.1.6. in July 2023 to Omicron E.G.5.1 in December 2023, with the epidemiology differing by geographical location (Figure 1). The current dominant variants have an immune evasion advantage, but result in less severe infections in the general population [39,48]. Here, more observation is needed to determine how variant transmission impacts the IEI community in terms of infection rates and severity of illness.

## 2. COVID-19 Vaccines and Vaccine-Induced Responses in Healthy Individuals

The emergence of SARS-CoV-2 and the rapid roll-out of new vaccine formulations have provided a unique opportunity to examine vaccine responses in infection- or vaccine-naïve individuals with or without IEI. This has the capacity to provide novel insights into the immunological defects in individual IEI patients [19], as well as to provide crucial information to direct future vaccination programs.

### 2.1. Vaccine Formulations for COVID-19

During the pandemic, the WHO evaluated and provided Emergency Use Authorization (EUA) qualification for COVID-19 vaccines in July 2020, with the initial aim to provide protection from viral transmission, severe disease, hospitalization, and death [51,52]. As viral transmissibility increased (September 2020), the focus switched to the latter aspects of vaccine efficacy, as well as the predominance of breakthrough infections [2,53]. To date, more than 13.6 billion doses of COVID-19 vaccine have been administered in the ongoing fight against SARS-CoV-2. This includes receipt of at least one dose of COVID-19 vaccine by 70.6% of the world’s population and 32.9% of people in low-income countries [1]. This, in isolation, shows the disparity in vaccine access between low and middle/high income countries. Granular data are not available in many regions to assess the extent to which this disparity impacts people with IEI.

The vaccine formulations used for primary dosing in Australia, the UK, and the USA included mRNA vaccines (Comirnaty (BNT162b2, Pfizer BioNTech)), SpikeVax (mRNA-1273, Moderna), viral vector vaccines (Ad26.COV2.S (Johnson & Johnson, Janssen), adenoviral vector based ChAdOx1 nCoV-19 (Oxford/AstraZeneca)), and one protein subunit vaccine (Novavax, Nuvaxovid). All vaccines are generally safe and highly effective at preventing hospitalization in healthy populations. Notable exceptions included the increased incidence of thrombosis with thrombocytopenia syndrome or severe anaphylactic reactions to polyethylene glycol in certain individuals with specific vaccine formulations [54,55]. Importantly, these five authorized COVID-19 vaccines induce a robust humoral response in healthy individuals, with some variation in antibody levels identified between different formulations as well as based on timings between doses [53,54,56]. For example, it has been shown that spike RBD-specific IgG antibody levels are higher in healthy individuals who received two primary doses of the Comirnaty mRNA vaccine compared to those who received two primary doses of the ChAdOx1 nCOV-19 adenoviral vaccine [5,6,57,58]. The development and rollout of the primary immunization series, and any subsequent boosters, are now an indispensable cornerstone of the global response against COVID-19.

In comparison, other authorized vaccination regimens, including Coronavac (Sinovac Biotech), Sputnik-V (Gam-COVID-Vac), and BBIBP-CorV (SinoPharm, Beijing Institute Biological Products), produce a substantially reduced antibody response with seropositivity rates of 36.9–50%, known to be substantially lower than those seen in convalescence. This explains the reduced efficacy of these vaccines with respect to both protection from symptomatic infection and severe infection and hospitalization [59,60,61].

Despite the emergence of SARS-CoV-2 variants as early as May 2021, most COVID-19 vaccine formulations until late 2022/early 2023 exclusively contained the spike protein of the ancestral WH1 strain. To combat ongoing viral evolution, bivalent mRNA vaccines were developed and released to further boost immunity to emerging Omicron variants, with the aim of providing protection from severe disease, hospitalization, and death. To date, four bivalent vaccines have emerged: the Pfizer bivalent vaccine containing equal amounts of WH-1 and Omicron BA.1 (Comirnaty Original/Omicron BA.1) or Omicron BA.4/BA.5 spike (Comirnaty Original/Omicron BA.4-5) [62] and the Moderna bivalent vaccine containing equal amounts of WH-1 and Omicron BA.1 (Spikevax Bivalent Original/Omicron BA.1) or Omicron BA.4/BA.5 spike (Spikevax Bivalent Original/Omicron BA.4-5). In May 2023, due to the increased prevalence of Omicron XBB.1 lineages, the WHO recommended use of the monovalent XBB.1.5 as the antigen for ongoing COVID-19 vaccination. Since then, two monovalent Omicron XBB.1.5 vaccines, Pfizer (Comirnaty) Omicron XBB.1.5. and Moderna (Spikevax) Omicron XBB.1.5, have been approved for use by the U.S. Food and Drug Administration (FDA), Australian Technical Advisory Group on Immunization (ATAGI), and the UK medicines regulator [15,63,64].

Due to the sparsity of information regarding the efficacy of bivalent vaccinations in IEI populations, this review summarizes the information to date on the healthy individual and IEI patient response to the original monovalent vaccination formulations.

### 2.2. The COVID-19 Vaccine-Induced Response in Immunocompetent Individuals

In immunocompetent individuals, COVID-19 vaccination generates neutralizing antibodies, as well as functional SARS-CoV-2-specific B-cell memory (Bmem) and CD4^+^ and CD8^+^ T memory (Tmem) responses, namely towards the RBD known to directly bind the ACE2 receptor. These correlates define a protective immune response emerging 1–2 weeks following vaccination. Whilst specific antibody and neutralizing titers have been shown to wane 1–3 months post-vaccination, protection against severe infection has been shown to persist for at least 4–6 months. Importantly, vaccine-induced Bmem and Tmem responses are known to persist longer-term, and likely represent a more durable marker of immune protection than antibodies [5,6].

Moreover, it has been shown that booster vaccination can significantly boost Bmem responses specific for ancestral virus and are cross-protective against Omicron subvariant RBD [56,57,58]. It is important to note that vaccine formulation can have a dramatic impact on the magnitude of response, such that individuals who received a two-dose primary regimen of Pfizer BNT162b2 generated a significantly higher magnitude of ancestral WH1 RBD-specific plasma IgG and Bmem compared to those receiving the two-dose adenoviral ChAdOx1 vaccine [56,58]. However, upon boosting with a third dose of mRNA vaccine, both groups demonstrated a significant increase in ancestral WH1 RBD-specific plasma IgG and Bmem numbers, with the magnitude of response being similar between both groups. Furthermore, the mRNA third-dose booster increased recognition of the Omicron BA.2 and BA.5 variants by both antibodies and Bmem [56]. This further exemplifies the need to assess these attributes of the immune response in COVID-19-vaccinated IEI patients and to determine whether the boosters have the same benefit in this population.

## 3. The COVID-19 Vaccination Response in IEI Patients

Globally, it has been acknowledged that immunocompromised individuals, including those with IEIs, may benefit from extra doses of COVID-19 vaccines to generate responses equivalent to those observed in healthy individuals [65,66,67,68]. First, it was recommended by the U.S. FDA, ATAGI, and UK medicines regulator that an additional (third) dose be added to the primary regimen. This differs from the two-dose regimen (except for Ad26.CoV2.S, which comprised a 1-dose regimen) for the general population [55,67,69]. Vaccines approved by the aforementioned regulatory bodies showed evidence of a high efficacy for protection from infection, as well as protection from severe disease, hospitalization, and death [54,70]. Whilst breakthrough infections with SARS-CoV-2 variants have been documented in patients with IEIs, those who have been vaccinated experience lower disease severity [71].

**Figure 2:**
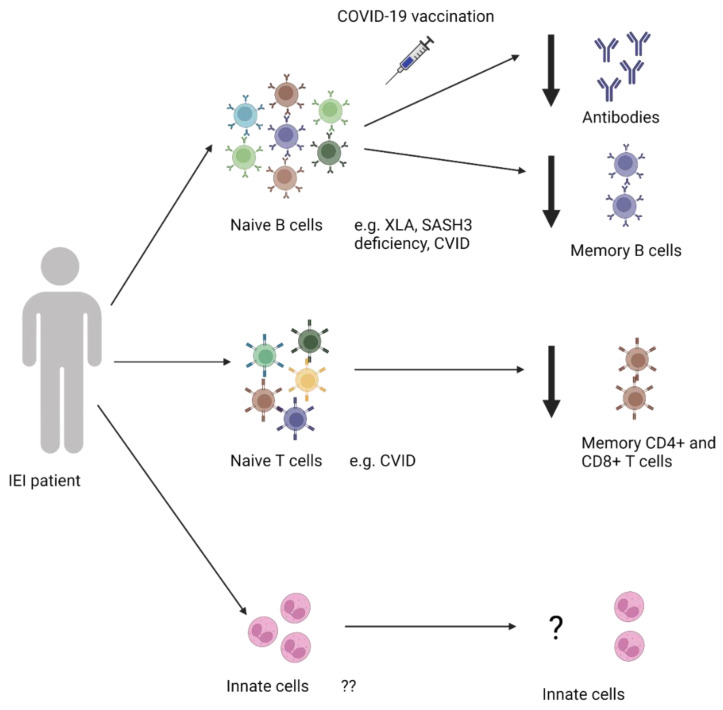
Generation of SARS-CoV-2-specific immunity in responses to COVID-19 vaccination in IEI patients. In healthy individuals, after administration of the COVID-19 vaccination, naive B- and T-cells are primed and expand to generate long-lived memory specific for the SARS-CoV-2 spike protein contained in the vaccine. This includes spike-specific antibodies, memory B cells, and memory T-cells that are ready for action upon re-exposure to antigen because of either booster vaccination or natural infection. In contrast, IEI patients are reported to generate a variable and, in some cases, poor response to COVID-19 vaccination which is dependent upon the genetic defect they possess. In most studies, COVID-19 vaccination of IEI patients can result in reduced COVID-19-specific antibody, memory B-cell, and/or memory T-cell responses, dependent upon the nature of the genetic lesion and the arm of the immune system affected. Above, we indicate IEIs for which perturbed COVID-19 vaccination responses have been reported in the literature. Additionally, innate cells play a pivotal role in the first line of defense against viruses. However, the responses of these cells to vaccination are currently unknown. ? depict unknown response and downwards depicts known reduced response. Figure generated with BioRender.

In contrast to the general population, individuals with IEI have variable immunological responses to vaccination, with responses being impaired in some patients [72,73,74,75,76,77] (Figure 2). This section will review the humoral response reported in the literature in a variety of IEIs. For the purposes of this review, we refer to doses 1–3 as the “primary” series for people with IEIs and any additional doses as “boosters”. This differs from healthy individuals, where the “primary” series refers to doses one and two, with doses three and onwards qualifying as boosters.

### 3.1. Humoral Response—Antibodies

**Figure 3:**
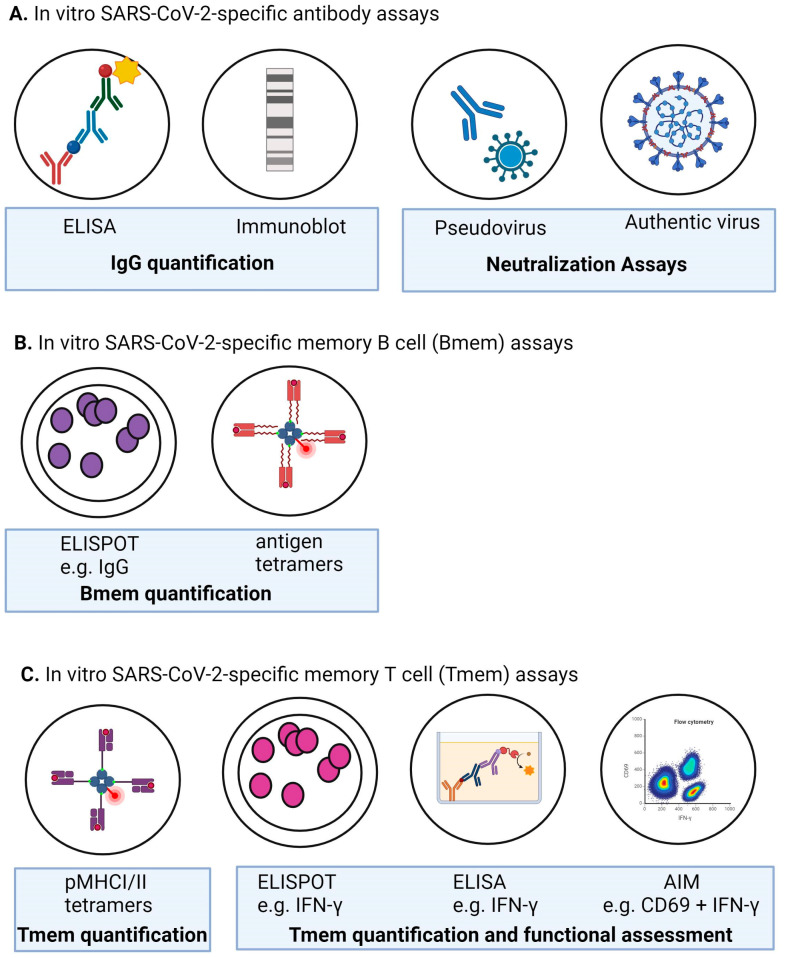
Main methods for the in vitro detection of SARS-CoV-2-specific humoral and cellular immunity in the peripheral blood of humans. (**A**) SARS-CoV-2-specific antibodies can be detected by ELISA or immunoblot, whilst SARS-CoV-2 virus neutralization is most frequently quantified using pseudovirus or authentic virus neutralization assays. (**B**) SARS-CoV-2-specific memory B cells (Bmem) can be identified either by the capture of IgG after antigenic stimulation in an ELISPOT or using fluorescently labeled SARS-CoV-2 antigen containing fluorochromes. (**C**) SARS-CoV-2-specific memory T-cells (Tmem) can be identified either by binding to fluorescently tagged peptide loaded MHC tetramers or after stimulation with whole protein or overlapping peptide pools. Here, SARS-CoV-2-specific Tmem are enumerated by either the production of cytokines such as IFN-γ in ELISPOTs or ELISAs or intracellular detection of cytokines coupled with cell surface expression of activation-induced markers (AIM) such as CD69, CD134, and CD137. Together, multiple methods have been used to assess immune responses to vaccination, preventing a robust comparison between various published reports (Table 2). Figure generated with BioRender.

People with antibody deficiencies generate a particularly poor antibody response to vaccination; indeed, “diagnostic vaccination” is used to clinically evaluate individuals for the presence of humoral IEI [78]. It was, therefore, an early concern whether these populations would develop any meaningful protection from COVID-19 vaccines, especially those using novel mRNA vaccine platforms. Here, we review 58 papers evaluating the humoral immune response generated by individuals with different IEIs to COVID-19 primary vaccines and/or booster doses (Table S2, Figure 2). In many cases [72,79,80,81,82,83,84,85,86], studies age-matched IEI patients to healthy controls to compare the robustness and quality of their antibody responses to those of the general population. This is important because otherwise, known age-related changes in the immune system would confound the results of each study. The published study designs varied from observational [74,80,86,87,88,89,90,91,92,93,94,95,96,97,98,99,100] to interventional studies and clinical trials [83,85,86,101,102]. Furthermore, humoral responses were reported according to seroconversion, antibody concentration/titers, and, in some cases, virus neutralization/pseudo-neutralization capacity (Figure 3 and Table 1). Samples were generally collected between approximately two weeks to one month post-vaccination, with a smaller portion of studies collecting samples at two, three, or six months post-vaccination [91,92,103]. These differences in sampling time post-vaccination can have dramatic effects on the status of the immune response. For these reasons, care should be taken when comparing studies, as variations in sample collection time points and experimental methodology affect the quantitative ranges, etc. (Figure 3 and Table 1).

Overall, seroconversion in populations with IEIs was much more variable and occurred at a lower magnitude compared to healthy controls across the primary series and additional doses [77,79,80,81,86,97,100,103,104,105,106,107,108,134,145,146,147], regardless of different methods of measurement. This is due to the heterogenous genetic and immunologic phenotype of disease in these individuals. In one study, individuals with IEI overwhelmingly displayed poorer seroconversion rates (ranging from 16.6–61.4%) compared to healthy controls (90–100%), after two doses of the mRNA vaccine (Comirnaty, SpikeVax) [76]. Despite the lower seroconversion rate at post-dose 2, it is clear that booster doses increased seropositivity (76–100%) in IEI patients after 3 vaccine doses [76,77,80].

As described in Section 1.1, the immune defects in patients with IEIs are highly diverse and likely impact the outcomes of COVID-19 vaccination. Most patients examined in the literature had PAD, in line with this group of diseases being the most prevalent IEI group (42% worldwide IEI diagnoses) [18]. In those receiving an mRNA vaccine, 20.6% to 90.0% of individuals with PAD seroconverted after two doses [74,77,84]. In contrast, in a cohort of individuals with primary or secondary antibody deficiencies vaccinated with either Comirnaty or ChAdOx1 nCoV-19, the overall seroprevalence rose from 61.4% to 76.0% after a third dose [76]. Tandon et al., 2023, assessed the differences between an adult (n = 62) and relatively smaller pediatric (n = 13) PADs cohort, finding that antibody levels were higher in the pediatric cohort after a primary series (Comirnaty), as well as after a monovalent booster, but only for those classified as having mild PADs [109]. Otherwise, there was limited research on pediatric PAD cohorts. A study by Shin et al. specifically investigated the clinical and/or immunological factors in a group of mixed PADs that might predict their COVID-19 vaccination response [110]. They found a lower seroconversion rate and neutralizing antibody levels to vaccination in CVID patients compared to other PAD categories (e.g., IgG deficiency, IgG2 subclass deficiency, and specific antibody deficiency (SpAD)). This was associated with a number of factors, including lower baseline IgG, IgA, and proportion of Bmem cells; a higher proportion of CD8^+^ Tmem cells; and a co-diagnosis of an autoimmune disorder [110]. Mizera et al. also found that, in their cohort study of various PADs, all patients in their CVID subgroup had around fivefold lower titers than other subgroups [108]. Thus, as stated by Quinti et al., with the high immunological and clinical diversity within CVID, each patient should be assessed individually for their capacity to mount a protective vaccination response [111].

In contrast, several studies have reported on smaller groups of patients with specific IEI diagnoses. For example, one report of individuals with STAT1-GOF (n = 7) found that 57% seroconverted after two doses of the vaccine [112]. Another case report on one individual with XMEN/MAGT1 deficiency reported a reactive spike-specific total antibody response after two doses of the mRNA vaccine [93]. In addition, Timothy et al. also completed a retrospective chart review of eight patients with CTLA-4 deficiency (two children and six adults). Of those who had their SARS-CoV-2-specific IgG levels tested (n = 2), antibody titers were lower (<6.25%) than those of the healthy unvaccinated controls [113]. Multiple studies evaluated antibody responses in individuals with XLA. In all studies, patients had nearly nonexistent antibody responses after two doses with either ChAdOx1 nCoV-19 or Comirnaty [84,102]. In fact, in one case study of three people with XLA, only one was able to mount a measurable antibody response post-vaccination [114] (Figure 2). This is consistent with the inherent B-cell defects in these individuals, whereby peripheral B cells are low or absent, resulting in an absence of serum immunoglobulins and an inadequacy in terms of generating a B-cell response to vaccination [16,17]. In this case, the observed post-vaccination antibody response is likely due to the IgRT received by the patient to treat their underlying antibody deficiency (See Section 3.1).

In several studies with more diverse cohorts of IEI, seroconversion differed by subgroup. Van Leeuwen et al. found significantly lower seroconversion rates in those with CVID, XLA, and CID compared to SpAD, undefined antibody deficiency, and phagocytic defects [74]. In studies which evaluated possible predictors of antibody responses in IEI populations, certain factors, such as condition severity [81,83], previous history of autoimmunity [72], underlying immunosuppressive treatment [81,83], and low numbers of Bmem cells pre-vaccination [84], were associated with poor responses (Figure 2).

This is important, as previous studies have established a connection between the humoral and cellular response to infection outcomes in healthy individuals [115].

Interestingly, in some studies which compared both COVID-19-naïve individuals and those with a history of previous SARS-CoV-2 infection, recovery from natural SARS-CoV-2 infection corresponded with a more efficacious antibody response to the vaccination series (i.e., “hybrid immunity”). This includes a more robust seroconversion rate [101,102], higher neutralization capacity [79], and better protection against the Delta variant [116]. Additionally, Abella et al. found that, even in the cohort with plasma cell diseases, those with multiple myeloma who had hybrid immunity had a higher plasma neutralization capacity than infection-naïve individuals 3 months post-vaccination [117]. This provides insights into whether hybrid immunity provides a more robust immune response than vaccination alone, in addition to the capacity of natural infection to shape immunity.

Recently, Abo-Helo et al. found that antibody levels remained poor in people with more severe disorders of humoral immunity 5–6 months after vaccination, suggesting the need for additional doses to preserve sufficient protection [85]. Van Leeuwen et al. conducted a large multicenter study of patients with PADs, following them up to 6 months past the primary vaccine series and a through a third dose for smaller subset of 50 CVID patients. Their cohort all received mRNA-1273 for their primary series, and either BNT16b2 or an unspecified vaccine formulation for a third dose, if applicable. At 6 months past the primary series, the decline in antibodies ranged from 5.9- to 11.2-fold, measured in GMT of S-specific IgG. However, seropositivity rates at 6 months ranged from 73% to 98%, with those in the XLA subgroup maintaining a relatively low rate of 24% [148]. Another study of a mixed cohort of more than 50 patients who received at least one dose of any mRNA-based OR inactivated vaccine, or a combination of both, found no significant difference in antibody levels across formulations [149].

There remains limited knowledge about the efficacy and durability of responses to different COVID-19 vaccine formulations in people with IEIs. Leung et al. studied the humoral response to both Comirnaty and CoronaVac (“Sinovac”) formulations in their cohort of mixed IEIs and found that 73% of individuals were seropositive after a full series of either formulation [96]. Some studies found that a full Comirnaty vaccination series was more efficacious than ChAdOx1 nCoV-19 [131], and this superior protection was maintained for about 6 months after the second dose [76]. Overall, most individuals had increased neutralizing activity after a partial or full vaccine series [72,73,74,76,86,96,102,116,131]. Furthermore, a follow-up from the COV-AD study using a live virus neutralization assay with Vero cells found that 99.8% of their study subjects had neutralization activity after a booster dose [102]. However, whether this was a bona fide response to vaccination or a consequence of receiving SARS-CoV-2-specific antibody-containing IgRT was not evident, although in at least one study, IgRT did not appear to be significantly associated with either titer or neutralization activity [77]. It is also important to note the sparsity of data surrounding SARS-CoV-2-specific Bmem generation in individuals with IEIs in response both to natural infection and SARS-CoV-2 infection, which is necessary to provide information about the generation and durability of the Bmem cells in these populations and their ongoing ability to produce antibodies.

Furthermore, to date, some IEI patients may have received five or more vaccine doses; however, information on the efficacy of these later doses is limited. This is likely due to lack of available participants with sufficient time elapsed since their most recent dose [102], and also insufficient time elapsed to enable sample analysis and reliable reporting of findings.

### 3.2. Memory T-Cells

Like humoral immunity, cellular immunity in people with IEIs is more variable than it is in healthy populations. Recent research has focused attention specifically on the mechanisms of Bmem, Tmem, and innate immune cells following COVID-19 vaccination in IEI populations.

As such, we reviewed 43 papers investigating the overall cellular response and its relationship with the humoral response of IEI populations after receiving COVID-19 vaccination (Table S3). As with antibody production, IEI patients demonstrated suboptimal CD4^+^ and/or CD8^+^ T-cell responses compared to the general population, ranging from ~30 to 80% of that of healthy controls [73,80,96,102,142,150]. A number of studies found that individuals with IEI could mount measurable CD4^+^ and/or CD8^+^ T-cell responses [89,106,107,143], measured as the percentage of IFN-γ-positive T-cells via IFN-γ release assay (IGRA), ELISpot assay, or flow cytometry (Figure 2, Table 1).

Studies evaluating both cellular and humoral post-vaccination immunity have found varying levels of correlation between these two responses, making it difficult to draw definitive conclusions about their relationship [96,99,101]. Sauerwein et al. found an association between intact spike-specific CD4^+^ T-cell responses and normal IgG responses, defined as a titer of spike protein 3x the limit or more, in individuals with CVID or milder antibody deficiency [134]. Oyaert et al. found a positive and significant correlation between anti-spike IgG and IFN-γ levels in IEI and chronic kidney disease (CKD) subgroups [103]. Notably, Gao et al. were able to confirm a positive correlation between antibodies and spike-specific CD4^+^ T-cells in all their study groups, both healthy and immunocompromised [141].

Other studies found that a negative humoral response was associated with a poor cellular response. Bergman et al. found that low levels of naïve CD4^+^ T-cells correlated with a poor antibody response in the CVID participants of their prospective clinical trial [79]. Similarly, Antoli et al. established that low CD4^+^ and CD8^+^ T-cell counts were a predictor of poor antibody response specifically in CVID patients [88]. Barmettler et al. also observed a correlation between low CD4^+^ T-helper cells and low specific antibody responses in their mixed cohort of individuals with secondary and severe primary PADs, including complicated CVID/SpAD, activated P13K-syndrome, TACI deficiency, NFKB1 deficiency, HGG, and IgG subclass deficiency. This observation was evident for both mRNA and the Ad26.CoV.S vaccines [82].

In contrast, several studies reported no association or a discordant relationship between the humoral and cellular responses [96,99,101]. For example, Pulvirenti et al. found nearly no T-cell response in their vaccinated cohort of people with CVID, despite this cohort presenting a positive (albeit suboptimal) IgG response [101]. Findings from a subsequent paper by Pulvirenti et al. demonstrate that T-cell abnormalities resulting from CVID may play a role in the lack of specific antibody responses after vaccination [94]. Interestingly, in a separate cohort followed by Goschl et al. had a robust specific CD4^+^ and CD8^+^ T-cell response to vaccination; they found no significant correlation between that response and antibody levels [128]. Similarly, a case study of five individuals with PAD by Steiner et al. found a robust cellular response, yet no humoral response in their entire cohort [151]. Mizera et al., in their study on a cohort of individuals with primary antibody deficiencies receiving 2–4 doses, found that anti-SARS-CoV-2 IgG did not correlate with either CD4^+^ or CD8^+^ levels, but rather NK cells [108]. This underscores the suggestion made by previous papers to measure both the cellular and humoral response in order to develop a more complete post-vaccination immune profile in each individual [152,153].

Importantly, some studies evaluated the spectrum of T-cell responses by IEI patients to identify specific characteristics or factors that may predict poor or robust responses. Shin et al. found a correlation between baseline immune profiles as well as comorbidities, where poor vaccine responders among their mixed cohort of PADs had autoimmune diseases, low levels of baseline IgG, low naïve CD8^+^ T-cells, and higher levels of effector CD8^+^ Tmem [110]. Gao et al., who looked extensively at T-cell profiles between subgroups of immunocompromised individuals, found that overall, specific CD8^+^ T-cells were generated at a higher rate than CD4^+^ T-cells. These frequencies were comparable at 35 days after dose 2 between healthy controls, IEI, HIV, and HSCT, but lower in SOT and CLL [141]. This study found that individuals enrolled in the trial generated higher levels of higher frequencies of SARS-CoV-2-specific CD4^+^ T-cells after two vaccine doses [141]. Similarly, van Leeuwen et al. conducted a multicenter study of different subgroups of IEI compared to healthy controls. Only the CVID subgroup had significantly lower IFN-γ response rates compared to healthy controls. Within this subgroup, lower responses were associated with age and lymphoproliferative diseases [74].

Similarly, we identified case reports of vaccination among less researched IEIs, including STAT1-GOF and X-linked *SASH3* deficiency, which found that nearly all studied individuals were able to mount both a cellular response and a humoral response despite a suboptimal baseline immune profile due to their inborn immune disorders [112,154] (Figure 2). Notably, Bloomfield et al. found that 85% of their STAT1-GOF cohort, including two individuals on the JAK inhibitor ruxolitinib, produced cellular responses comparable to healthy controls [112].

Like with post-vaccination antibody levels, exogenous factors such as natural SARS-CoV-2 infection and vaccine formulation appear to impact T-cell responses in certain settings. Some studies found that SARS-CoV-2 infection increased T-cell activity in antibody-deficient individuals [155], while another found poor CD4^+^ and CD8^+^ T-cell responses after both previous infection and subsequent vaccination [101]. Other studies have reported that prior SARS-CoV-2 infection results in improved immune response after the first [156] and second [102] vaccine doses. Additionally, other studies have reported the phenomena of pre-existing cross-reactive CD4^+^ T-cells that can recognize SARS-CoV-2 [157]. The COV-AD study follow-up found that receipt of heterologous vaccines by infection-naïve IEI individuals was associated with a significantly higher likelihood of T-cell response generation compared to homogenous boosting [76]. Lin et al. found that a third dose increased the amount of SARS-CoV-2-specific CD4^+^ and CD8^+^ T-cells generated [90], and Goda et al. were able to demonstrate a 30% increase in T-cell activity after a third dose of Comirnaty following two ChAdOx1 n CoV-19 in individuals with CVID [131].

The rise of viral variants has sparked questions about the durability of T-cells, as well as whether strain-specific T-cells are broadly protective against variants in the IEI population. While few studies have been published to date in either healthy populations or individuals with IEI, there is some evidence of sustained reactivity.

In terms of longevity, Hurme et al. conducted a longitudinal cohort study over 22 months in a mixed cohort of primary and secondary antibody deficiencies, finding a robust cellular response. Specifically, across 1–4 doses of either mRNA-1273, BNT162b2, or ChAdOx1, 95% of their cohort had detectable T-cell responses after the primary series as measured by flow cytometry, dropping down to 78% at 3 months following a fourth dose. In a smaller subset of individuals, they observed that CD4+ cell responses were higher than CD8^+^ at the same timepoints when stimulated with wildtype SARS-CoV-2 strain [105].

Van Leeuwen et al. extended their investigations to observe cellular responses at 6 months post-primary vaccination in their mixed IEI cohort, finding that while IFN levels decreased significantly in most of their subgroups (XLA, CVID, IgG/SPAD), these responses were still detectable at varying levels [148]. Barouch et al. found that the Ad26.CoV2.S vaccine induces durable cellular immunity, with minimal decreases over the course of 8 months in a healthy population [135]. In another healthy individual cohort, Liu et al. found that both the Ad26.CoV2.S and Comirnaty vaccines induced durable spike-specific CD4^+^ and CD8^+^ T-cell responses. Here, 82–84% CD8^+^ T-cell responses were cross-reactive to the Omicron B.1.1.529 variant [150]. Sanchez et al. recently demonstrated that people with XLA had increased T-cell responses to variants compared to healthy controls, while individuals with CVID had reduced responses [144]. More evidence is needed to understand the post-vaccine T-cell response in general, as well as its ability to provide protection in the face of future SARS-CoV-2 variants. This, coupled with correlation between generation and durability of Tmem and Bmem responses, will provide crucial information about whether, for example, the generation of Tmem responses in the absence of Bmem is sufficient for protection from disease.

### 3.3. Memory B Cells and Innate Cells

Very few studies have evaluated the post-vaccination production of Bmem or the innate immune response (Table S4). Those that have tended to concentrate on the correlation between the presence of these cells and SARS-CoV-2-specific antibody levels. Most studies reviewed found an association between measures of Bmem and humoral responses. In three studies following individuals with antibody deficiencies, their reduced antibody responses correlated with reduced Bmem count [79,88,110]. However, other studies found little to no correlation between B-cell measurements and humoral responses [101,154], likely due to patients receiving IgRT, where antibodies from the product are indistinguishable from vaccine-induced antibodies. As with the T-cell response, Lin et al. found a similar effect of a third vaccine dose, which increased SARS-CoV-2-specific B-cell responses [90]. The previously mentioned individual with SASH3 deficiency had marked B-cell lymphopenia and reduced switched Bmem cells [154]. Pulvirenti et al. found that SARS-CoV-2 infection produced a more classical Bmem cell response in unvaccinated individuals with CVID, whilst vaccine recipients with CVID had more atypical [CD19^+^CD27^−^CD24^−^CD38^−^] Bmem responses [101].

At the time of this review, there was limited literature regarding specific innate cell responses to SARS-CoV-2 primary vaccination in individuals with IEIs. Notably, Cuapio et al. recently conducted a clinical trial observing natural killer (NK) cells in IEI/SID individuals during COVID-19 vaccination. They found a positive correlation between the frequency of NK cells at baseline and antibody titers taken about a month after vaccination (2.1 T-cell section). Therefore, further elucidation of the innate cell response to vaccination will be important in terms of fully understanding the efficacy of COVID-19 vaccines in these populations [158]. Mortari et al., 2023, conducted a longitudinal study on their cohort of only CVID patients who received 1–4 doses of BNT162b2 and identified four functional groups of B cell phenotypes and clinical characteristics. With respect specifically to Bmem cells, they classified their cohort into those with both low- and high-affinity Bmem cells, those who only had low-affinity Bmem cells, those with neither low- nor high-affinity Bmem cells, and non-responders (neither Bmem cells nor specific antibodies). Overall, their CVID group had significantly lower levels of low- and high-affinity Bmem cells compared to healthy volunteers [145]. Steiner et al., 2023, in their smaller cohort of only CVID patients, found that after a primary vaccine series, those who did seroconvert had significantly lower levels of class-switched Bmem cells [107]. Finally, Yam-Puc et al. conducted interesting analyses of the role of age-associated B cells (ABC) in the vaccine responses of those with IEIs or cancer, finding that a higher baseline level of ABC is associated with lower levels of specific memory B cells and less neutralizing activity [147].

The reviewed literature highlights the important finding that, for certain IEI conditions, solely relying on measurements of antibody response as a proxy for overall immunity may be underestimating the durability and efficacy of COVID-19 vaccines [79]. Given the potential confounding factors of disease, IgRT, and other adjunctive therapies (see below), it will be important to devote future research initiatives to specifically evaluating the SARS-CoV-2-specific Tmem, Bmem, and innate immune cell responses to COVID-19 vaccination in IEIs to further clarify their efficacy in these populations. Furthermore, it is evident that there is a paucity of information defining what response level of each individual immune correlate is required to confer protection against SARS-CoV-2. This information is urgently needed to accurately discern whether COVID-19 vaccines generate protective responses in people with IEIs.

## 4. Impact of Adjunctive Antibody Therapies on COVID-19 Immunity

Antibodies that are generated against commonly encountered pathogens and vaccine antigens are readily present in IgRT, which reduces the infectious burden in the recipient of this therapy [22,25]. Considering the ongoing pandemic, the presence of SARS-CoV-2-specific antibodies in IgRT and its capacity to protect people with IEIs from infection and/or severe COVID-19 has been of great interest to patients, clinicians, and the scientific community. Furthermore, the development and rollout of monoclonal SARS-CoV-2 antibody (mAb)-based therapeutics as preventatives and treatment options for COVID-19 has been extremely pertinent (Table 2), particularly considering the known lag between infection (and, once available, vaccination) of the general population and emergence of these antibodies in IgRT. Here, we review our current understanding regarding the content and functional capacity of SARS-CoV-2 antibody-containing products and their ability to protect against circulating SARS-CoV-2 variants.

**Table 2:** Adjunctive therapies for prophylaxis or treatment of SARS-CoV-2 infection.

	Monoclonal		Polyclonal
Example	Evusheld (AZD7442)	Xevudy (sotrovimab)	IgRT (IVIg or SCIg)
Company	AstraZeneca	VUR Biotechnology GlaxoSmithKline	Various
Pre or Post Exposure Prophylaxis	Pre	Post	N/A
Antibody Isotype	Hu IgG1κ	Hu IgG1κ	Various
SARS-CoV-2 Target	RBD	RBD	Broad range including NCP and RBD
Origin/Platform	Isolated from B cells of convalescent patients	Natural antibody from convalescent patient with SARS-CoV-2	Purified antibodies from plasma of >1000 donors/product batch
Half-life	~6 months	LS mutation of Fc increases half life	~28 days
Doses required	1 *	1	ongoing
Dosage	150 mg of tixagevimab + 150 mg cilgavimab	500 mg	Dependent on factors including weight and IgG trough levels
Frequency of redosing	Every 6 months	N/A	Every week to every month depending on IgG trough levels
Route of administration	IM	IV	IV or SC
Approved recipients Age COVID-19 exposure Clinical disease	>12 years weighing at least 40 kg Not infected and no recent exposure Moderate to severe immunocompromised state	>12 years weighing at least 40 kg COVID-19^+^ PCR Moderate to severe immunocompromised state	Dependent on established clinical guidelines
Neutralization Omicron BA.1 BA.2	344-fold decrease 9-fold decrease	Unknown Unknown	Unknown Unknown
Emergency Authorization US FDA Australian TGA	12/21–1/23 11/21–1/22	05/21–3/22 08/21–3/22	N/A
Emergency approval for use in	Australia, UK, and USA	Australia, UK, and EU	N/A
References	[159,160,161]	[55,160,162,163]	[164]

COVID-19, coronavirus 19; FDA, Food and Drugs Administration; Hu, human; IgRT, immunoglobulin replacement therapy; IM, intramuscular; IVIg, intravenous immunoglobulin; subcutaneous immunoglobulin, SCIg; N/A, not applicable; nucleoprotein, NCP; receptor-binding domain, RBD; severe acute respiratory coronavirus-2, SARS-CoV-2; TGA, therapeutic goods administration (* Dose increased to 300 mg each antibody in 12/22 due to emergence of Omicron BA.1 and BA.1.1 [165]).

### 4.1. Immunoglobulin Replacement Therapy (IgRT)

IgRT is the cornerstone of treatment for antibody-deficient individuals. For over 25 years, prophylactic administration, either intravenously (IVIg) or subcutaneously (SCIg), has been shown to be safe and tolerable, reducing the incidence and severity of infection [21,22,164]. IgRT is a polyclonal IgG product purified from pooled human plasma (>1000 donors) containing a broad range of antibody specificities directed against commonly encountered pathogens, either because of natural infection or vaccination in the general population (Table S5) [164]. However, when SARS-CoV-2 first emerged, there was a period of multiple months before IgRT products contained IgG against SARS-CoV-2. This gap in protection was problematic due to the reliance of antibody-deficient individuals on the protection this product provides against a variety of infections. It was believed that this gap in protection would compound the immune defects already predisposing immune-deficient individuals to severe COVID-19 disease.

However, even when SARS-CoV-2-specific antibodies from natural infection and, later, vaccination in plasma donors emerged in IgRT, it was unclear whether they would neutralize the virus in recipients. Moreover, early insights into the protective capacity and persistence of SARS-CoV-2-specific neutralizing antibodies (nAbs) in plasma from convalescent COVID-19 patients showed that these antibodies could protect from viral transmission and remained stable in plasma from 15 months post-infection while retaining their capacity to protect from infection [5,6,23]. This provided an avenue for treatment for at-risk individuals who contracted the virus, including those with IEIs, to both limit infection and protect from severe disease. This also provided promise that, when antibodies emerged in IgRT, they would provide some protection against SARS-CoV-2 [23,118,119]. Despite this, it was necessary to obtain data to prove the retention of antibody function after the IgRT manufacturing process.

We reviewed 21 papers (Table S4) documenting the occurrence of SARS-CoV-2-specific antibodies in products derived from plasma donations mainly from Europe and the USA. At the time of printing, information regarding SARS-CoV-2-specific antibody content in other countries, including Australia, was not available. Observational studies showed that products generated from pre-pandemic plasma contained antibodies which were cross-reactive to seasonal coronaviruses and SARS-CoV; however, none demonstrated the capacity to neutralize the SARS-CoV-2 virus [120,121,122,123,136]. A study by Farcet et al. first detected SARS-CoV-2-specific antibodies in products generated from USA-donated plasma in September 2020 [137], and Romero et al. documented the first positive pools from donations in Spain and the USA in July through September of the same year [138]. A later update by Farcet et al. showed that neutralization towards the ancestral WH1 strain plateaued in products between May 2021 and April 2022, with the drop in titers in European plasma being slower compared to the USA [139], likely due to changes in infections rates in these locations. Importantly, antibody titers in IgRT products steadily increased longitudinally with increasing incidence of SARS-CoV-2 infection, with 93% of IgRT lots containing SARS-CoV-2-specific antibodies by January 2021 [126,137]. A later study by Raphael et al. documented a twofold increase in SARS-CoV-2-spike-specific IgG antibodies in products between February and March 2021. Importantly, this paper associated products with high antibody titers with donations from countries with high rates of COVID-19 infection and products with low titers with countries with low infection rates [166]. Additionally, while the published studies clearly document the emergence of anti-spike SARS-CoV-2 IgG levels [119,120,121,122,123,124,125,126,127,137,138,139,167], in a few cases, nucleocapsid-specific IgG was also observed, resulting from natural infection of the donors prior to plasma donation [124,139,167]. This clearly demonstrates the association between geographical location, timing of plasma donations and natural infection, vaccination rates at the time of donation, and the subsequent emergence and levels of SARS-CoV-2 antibody in commercial IgRT.

Additionally, ongoing assessment of IgRT demonstrated that, while the recognition of ancestral WH1 remained high, cross-reactivity towards variants decreased incrementally with continued mutation of the RBD of SARS-CoV-2. Here, the cross-recognition levels of the Omicron variant were lowest [130,133,168], mirrored by a decreased neutralization capacity and reduced inhibition of ACE2 receptor binding [133,168]. This is in line with observations from healthy donor plasma post-vaccination [56,57,58].

Neutralization capacity of antibody in IgRT to the ancestral SARS-CoV-2 strain was assessed in 11 publications, although multiple assays (Table 1) were utilized, making definitive comparisons between the neutralizing capacity of products between studies difficult [118,119,120,123,124,125,126,132,136,137,139]. Despite the continuing emergence of SARS-CoV-2 variants, few of these studies assessed the presence or neutralization capacity of antibodies to these variants. In one study in April 2022, all lots derived from US plasma could neutralize Omicron B.1.1.529, albeit at a 12-fold lower level compared to the ancestral strain and a 22-fold lower level in donated plasma from Europe [139]. A second study showed 32-fold lower Omicron neutralization compared to the ancestral strain [126]. A third study demonstrated neutralization of Omicron BA.1, BA.4/5, BQ.1.1, and XBB, with these antibodies retaining neutralization capacity in the plasma of patients receiving these products [130]. Notably, the latter study is the only one to date that tested the product administered to each patient alongside their plasma both pre- and post-infusion. This enabled the dissection of whether detected antibodies were endogenously produced in response to vaccination or were due to antibodies contained in IgRT. Importantly, this tracks the IgRT products’ capacity to retain its function within recipients and infers that these products play a role in limiting severe COVID-19 disease. Additionally, neutralization of ancestral and omicron variants was boosted after receipt of the product, further supporting the importance of this treatment for protection against SARS-CoV-2 infection. This, again, reflects the dynamics of the timing and levels of natural infection and vaccination in different geographical locations where plasma is donated. It also shows the decreased antibody recognition of variants, which has also been documented in studies examining antibodies induced by COVID-19 vaccination [57,58]. This observed reduction in RBD-specific antibody binding to the Omicron B.1.1.529 variant was also observed in serum from convalescent COVID-19 patients and COVID-19-vaccinated individuals [169]. These observations led to concerns regarding the ongoing efficacy of antibody-based therapies in the treatment of COVID-19.

The progressive loss of variant recognition and reduced neutralization potency is indicative of viral evasion from antibodies. These variables make it difficult to infer the level of protection IgRT confers towards breakthrough infection, much less the clinical dose that is efficacious in preventing severe disease. These facets will require further examination to provide valuable information to clinicians regarding clinical dosing with IgRT and the clinical need for other supportive treatments, including mAbs and antivirals, for the treatment of COVID-19.

### 4.2. Prophylactic Monoclonal Antibody (mAb) Therapies to Prevent Severe COVID-19 in IEI

Monoclonal antibodies were developed as a treatment option for immunocompromised individuals who were deemed highly susceptible to severe COVID-19, including individuals with IEIs and those who had received solid organ or hematopoietic stem cell transplants. Up to April 2022, a dozen mAbs targeting the RBD spike protein were rapidly developed and deployed for clinical use as a preventative against SARS-CoV-2 infection, or for treating those infected or recently exposed to SARS-CoV-2 to reduce the chance of severe disease, hospitalization, and death [170].

mAbs consisted of either single (e.g., sotrovimab or bebtelovimab) or combination therapies (e.g., Evusheld) aimed at reducing or limiting infection by preventing spike protein binding to the ACE2 receptor and targeting the virus for elimination either by direct neutralization, antibody-dependent-cellular phagocytosis, antibody-dependent T-cell-mediated cytotoxicity, or complement activation. While antibodies targeting other domains of the spike protein or other viral proteins are in development, here, we will review the literature surrounding the efficacy of sotrovimab and Evusheld (Table S5), the mAb modalities most prominently used as preventatives or therapeutics for SARS-CoV-2 in Australia, the UK, and the USA. We discuss their efficacy towards the ancestral WH-1 virus and VOC (Table S6), as well as future iterations currently in development to combat the ongoing emergence of VOC.

#### 4.2.1. Sotrovimab

Sotrovimab (Xevudy, VUR Biotechnology GlaxoSmith Kline) is a single-dose mAb administered intravenously for the treatment of SARS-CoV-2, and was first authorized for emergency use by the FDA in May 2021 (Table 2) [171]. In people treated with sotrovimab, antibody titers of the drug have been proven to be high at three months post-infusion [172]. This mAb has proven efficacy to the ancestral WH-1 strain of SARS-CoV-2 and the strains preceding Omicron, as evidenced by reduced COVID-19 hospitalization rates in individuals receiving treatment [173,174]. Separately, modeling studies have shown that sotrovimab’s efficacy could be predicted by the clinical stage of the disease at treatment, with treatment during early disease yielding better therapeutic efficacy [175]. However, the emergence of Omicron sublineages has been suggested to reduce mAb efficacy. Sotrovimab has been shown to maintain its efficacy in reducing the progression of breakthrough infection with Omicron BA.1 and BA.2, as evidenced by reduced hospitalization and/or death rates within 28–30 days of mAb administration [173,176,177,178,179,180]. Importantly, both subvariants showed a similar risk of hospital admission [179,181]. In contrast, a separate study showed no protective effect of sotrovimab against BA.2 [182]. Here, measurement of sotrovimab-treated individuals showed that neutralization of BA.1 was 4-fold lower than for BA.2, demonstrating a dramatic loss in spike protein recognition [181]. Furthermore, in murine models, sotrovimab administration to mice expressing the ACE2 receptor showed reduced lung infection with either Omicron BA.1 and BA.2. This illustrates that, despite reduced in vitro neutralization capacity, this mAb retained protective capacity against severe lung disease [183].

Continuous evolution of Omicron has been associated with ongoing immune evasion [5,6]. Here, the progressive loss of the neutralization efficacy of sotrovimab has been observed starting with the Omicron BA.1 lineage, with a full loss of efficacy predicted from BA.4.6 [183,184,185,186,187,188,189]. To date, Omicron BQ.1.1.10, BA.4.6.3, XBB.1.1, XBB.1.5, and CH.1.1 are the most antibody-evasive strains, with sotrovimab exhibiting reduced neutralization and binding to ACE2, resulting in increased viral transmissibility [190,191,192]. This demonstrated loss in efficacy resulted in a retraction of the authorization of sotrovimab use for the treatment of COVID-19 [193]. It is important to note that cell lines used for neutralization varied in their expression of ACE2, making the comparison of neutralization data between studies difficult (Figure 3, Table 2).

#### 4.2.2. Evusheld

Evusheld (AZD7442) is a mAb combination of cilgavimab and tixagevimab, which both bind to non-overlapping epitopes on the SARS-CoV-2 RBD, causing direct neutralization of the virus [194]. These antibodies, initially isolated from previously infected individuals, were engineered to extend their half-life (Table 2). Phase I trials showed that intramuscular administration of 300 mg Evusheld resulted in 10-fold higher levels of neutralization 3 months post-administration, with levels remaining above those of convalescent serum at 9 months. Furthermore, antibodies were detectable in nasal mucosa, a SARS-CoV-2 infection site, which might help to limit infection [194]. Furthermore, non-human primate models of SARS-CoV-2 showed that infection was prevented by prophylactic administration of Evusheld, while accelerated viral clearance was achieved by therapeutic administration [194]. Therefore, while this mAb combination was the only approved pre-exposure prophylaxis for COVID-19 in some countries [172,189,195], other countries, including Europe and Japan, approved Evusheld solely as a treatment of COVID-19. This demonstrates regulators’ dogmatic focus on not licensing COVID-19 mAbs for simultaneous prophylactic and treatment purposes [189].

Antibody levels in individuals receiving Evusheld were high 3 months post-administration, with breakthrough infections rare in recipients [172]. As of the Delta surge, increased serum antibody levels and efficient neutralization of the Delta variant were observed in all individuals receiving Evusheld [196]. In comparison, neutralization was decreased 344-fold in Omicron BA.1 and 9-fold in BA.2, with 13.8% of Evusheld recipients exhibiting breakthrough infections with these subvariants [196]. This was confirmed in vitro, where neutralization capacity was shown to be significantly reduced in Omicron BA.1, with a high rate of BA.1 breakthrough infections observed in kidney transplant recipients treated with Evusheld [195,197]. In comparison, Evusheld retained strong neutralization capacity against BA.2 in vitro for at least 8 weeks post-administration [172,195]. This shows the importance of ongoing testing of mAb therapeutics for emerging variants.

What was evident for Evusheld is that cilgavimab demonstrated better efficacy against both BA.1 and BA.2 than tixagevimab, accounting for Evusheld’s retained efficacy [185,196]. The ongoing evolution of variants has resulted in a progressive loss of variant recognition by Evusheld, reducing its capacity to neutralize the SARS-CoV-2 virus and protect from infection. As of January 2023, the FDA has rescinded authorization for emergency use of Evusheld as a pre-exposure prophylaxis for COVID-19 in the US [198]. In response to the ongoing threat from variants, AstraZeneca has now registered and is recruiting for a Phase I/III study to test a new pre-exposure prophylaxis in the SUPERNOVA (Study Understanding Pre-Exposure pRophylaxis of NOVel Antibodies; NCT05648110) trial. It is noted that Phase I will investigate this therapy in healthy adults, with extension in to immunocompromised adults and adolescents in phase III [199,200].

Altogether, this highlights the importance of ongoing assessment of mAb recognition of emerging viral variants to determine neutralization capacity, as well as, thus, the efficacy of these therapeutics.

## 5. Discussion and Future Directions

The SARS-CoV-2 pandemic has shed light on the role that cellular and humoral immunity play in the control of SARS-CoV-2 infection and elicitation of robust vaccination responses. Importantly, it has shown that solely assessing antibody responses to natural infection and/or COVID-19 vaccination is insufficient for monitoring the efficacy and longevity of SARS-CoV-2-specific immunity, especially in people with IEIs [100], particularly as spike-specific antibody levels wane over time [5,6,23,57,58]. Additionally, IgRT and other antibody-based therapeutics confound the measurement of endogenous infection- or vaccination-induced antibodies. This highlights the urgent need for strategies to easily detect antigen-specific Bmem and Tmem responses, particularly as the assessment of antibody responses traditionally used to measure vaccine responses is confounded by IgRT administration. These facets of immunity work in concert to provide robust protection against infection, severe disease, and death. Thus, measurement of their quantity, quality and longevity is paramount for assessing vaccine efficacy and their potential to protect from severe disease in both healthy individuals and those with conditions impacting immune function [179,180].

At present, assays examining these compartments vary and are only available in the research setting, making it difficult to compare findings between individual studies. Translating these assays to the diagnostic setting requires the dedication of resources, including funding and further refinement and testing of methodology, to ensure they are robust, standardized, and high-throughput. The availability of these assays is particularly important for IEI, as the genetic and immunological complexity of these diseases make it difficult to draw clear conclusions for immune-deficient individuals collectively. As such, individualized assessment is urgently needed to uncover the depth of the defect, the role of the cellular response in the absence of humoral immunity, and the level of protection that may be afforded by each correlate of immune function, thus imparting information regarding the efficacy of COVID-19 vaccination. A prime example of this is in XLA, where T-cell responses play a more prominent role due to the absence of both antibodies and B cells. Comparatively, in disorders with both a T-cell and a B-cell defect (e.g., CVID) it is crucial for immunity to be generated by both compartments to account for defects in both arms of the immune system. Future studies should focus on more than one facet of the immune system in order to understand and correlate responses generated in different compartments and to provide a more holistic picture of each individual patient’s response to vaccination. Additionally, analyses such as those from Ngyuen et al. bring attention to the importance of population-level factors such as social determinants of health in determining patient outcome and disease risk. Future investigations should consider adding these key factors into the assessment of the IEI population’s response to COVID-19 infection and vaccination in order to provide a more wholistic understanding of what drives disease and protection in these populations.

There is currently a paucity of information regarding the number, function, and phenotype of Bmem and Tmem generated in response to COVID-19 vaccination in people with IEIs, as well as the capacity of these cells to bind and neutralize SARS-CoV-2 variants. This is important in the ever-evolving landscape of viral mutation, where even in healthy individuals, antibodies and Bmem cells exhibit reduced recognition of variants. In the context of IEI, reduced efficacy could render these populations more susceptible to severe disease and possibly death.

Finally, to date, assessments of vaccine responses in IEI patients have only been published for up to 3–4 doses of COVID-19 vaccines [76,82,87,90,94,96,131]. While this has provided critical information on early post-vaccination immune responses, immune-deficient individuals are recommended to receive boosters every 3–6 months. Thus, at present, some IEI patients have received six or more doses of the vaccine compared to 4–5 in the general population. Therefore, information regarding the efficacy of these later doses, especially those with the bivalent formulations, and protein-based vaccines (e.g., NovaVax) is required in order to determine whether certain vaccine platforms elicit a superior immune response and whether individuals with humoral deficiencies may benefit from a formulation such as NovaVax that specifically induces a T-cell response. This information is urgently needed to ascertain the benefit of continued vaccination and novel formulations in IEI populations. Together, this knowledge will inform future COVID-19 vaccine recommendations for people with IEI, and more widely, other immunodeficient populations. Additionally, these insights might also have implications for the regimens for other currently administered vaccines (e.g., influenza) and for pathogenic threats that may emerge in the future.

In summation, there is much that is left to understand about COVID-19 vaccinations in people with IEIs. These complex issues require intensive research to provide robust recommendations concerning timing between doses, vaccine formulations for individual disorders, and whether vaccines provide sufficient protection to allay the anxiety brought by increased transmission of SARS-CoV-2. In addition, it is important to generate continued evidence of the capacity of IgRT and mAb treatments to bind and neutralize emerging variants to inform the clinical care of people with IEIs as both prophylactic and treatment options for COVID-19. This ongoing pandemic is known to affect those with IEIs physically, mentally, and socioeconomically. We hope the information in this review and that of ongoing and future studies will benefit individuals with IEI, ultimately improving their quality-of-life post-pandemic.

### Final Thoughts

This review shows that COVID-19 vaccine responses in people with IEIs are highly variable after a primary vaccine regimen of three doses (compared to two in the general population). Whilst there is limited information on the responses of people with IEIs to booster doses of the vaccine, it is clear that this variability still exists. Furthermore, there is limited research on the capacity of cross-recognition of SARS-CoV-2 variants by immune cells in people with IEIs. Despite this, we can infer from information in the general population that repeated vaccination is required to boost immunity to Omicron variants [56], which is presumed to enable ongoing protection from severe disease.

However, in order to make specific recommendations for this population, further research is needed to accurately determine whether these same patterns are observed in people with IEIs, as well as to standardize the methodology for measuring antigen-specific immunity in peripheral blood. Research in three main areas is required. These are: 1. IEI patient responses to three or more doses of COVID-19 vaccine to provide evidence of protective immunity and to ascertain the requirement for continued administration of booster vaccine doses and their frequency. 2. The capacity of antibodies in IgRT products to recognize and neutralize circulating Omicron variants; this will demonstrate that these products provide some protection against severe disease in people with IEIs. 3. Further information on the severity and duration of COVID-19 disease upon infection and incidence of long COVID in IEI people to ascertain the vulnerability of this population to circulating Omicron variants, the efficacy of methods to limit disease, and the ongoing measures required to protect this population.

Once more of this information is available, then these data will prove crucial in drafting guidelines for vaccination of this population, not only for COVID-19, but also for other viral pathogens.

## Figures and Tables

**Table 1:** Methods used for the evaluation of cellular and humoral immune responses.

Assay	References	
	Studies Investigating	
	IEI Patient Responses to COVID-19 Vaccination	Quantity and Quality of IgG Antibodies in IgRT
SARS-CoV-2-binding IgG antibodies		
SARS-CoV-2 IgG II Quant Assay	[87]	-
Luciferase immunoprecipitation	[100]	-
ELISA	[82,83,84,86,92,94,97,103,104,105,106,107,108,109,110,111,112,113,114,115,116,117]	[118,119,120,121,122,123,124,125]
Abbott/Phadia ELiA SARS-CoV-2-Sp1 IgG assay	-	[126,127]
Roche Elecsys immunoassay	[80,82,83,104,128,129]	[130]
DiaSorin IgG immunoassay	[80,81,88,103,129,131]	-
Microblot assay	[112]	-
Abbot nucleoprotein assay	[81]	-
His-tagged spike and RBD binding probes	[90]	-
Abbott IgG assay	[85,91]	-
Luminex Ab binding	[98,124]	-
Extracted cell-free DNA sequenced by DNBSEQ	[92]	-
Abbott Quant chemiluminescent microparticle immunoassay	-	[132]
Abbot AdviseDx SARS-CoV-2 IgG II assay	-	
V-PLEX SARS-CoV-2 10-plex IgG		[133]
SARS-CoV-2-neutralizing antibodies		
SARS-CoV-2 Neutralization Antibody Detection Kit	[110,124,134]	-
Pseudovirus neutralization assay	[74,82,86,90,92,95,117,131,135]	[120,125,132,136,137,138,139]
ELISA	[72,73,89,96]	-
Live neutralization assays using Vero cells	[76,102,128]	[126]
B-cell measurement	[84]	-
Luminex based assays	[98]	[130]
SARS-CoV-2-specific B cells		
SARS-CoV-2-specific tetramers	[73,84,90,101,140]	-
SARS-CoV-2-specific T-cells		
AIMS (cell surface + intracellular cytokines)	[79,86,94,110,134,141]	-
Intracellular cytokine staining	[92,96,135,142]	-
Interferon-y ELISA	[88,89,102,103,143]	-
Interferon-y ELISPOT	[73,76,79,84,86,92,101,128,131,144]	-
Whole blood IGRA	[74,99,112]	-
Tetramers/multimers pMHCI		
	[142]	
pMHCII	[90]	

AIMS, activation-induced markers; DNBSEQ, DNA nanoballs sequencing; EliA, Enzyme fluoroimmunoassay; ELISA, enzyme-linked immunosorbent assay; ELISPOT, enzyme-linked immunosorbent spot; IgG, Immunoglobulin G; IGRA, IFN-γ release assay; pMHCI, peptide major histocompatibility complex I; pMHCII, peptide major histocompatibility complex II; SARS-CoV-2, Severe Acute Respiratory Syndrome Coronavirus-2.

## Supplementary Materials

The following supporting information can be downloaded at: https://www.mdpi.com/article/10.3390/vaccines12060675/s1, Table S1: Current and previous SARS-CoV-2 variants and their characteristics; Table S2: Antibody responses; Table S3: Memory T-cell responses, Table S4: Bmem and Innate responses; Table S5: IgRT; Table S6: Therapeutic mAbs. References [39,201,202,203,204,205,206,207,208,209,210,211,212,213,214,215,216,217,218,219,220,221,222,223,224,225,226,227] are cited in the Supplementary Materials.
