# Supplementary material for: An Overview of the Strategies to Boost SARS-CoV-2-Specific Immunity in People with Inborn Errors of Immunity
Source: Vaccines (Basel). 2024 Jun 18;12(6):675. doi: 10.3390/vaccines12060675 (PMC11209597; doi:10.3390/vaccines12060675)
Supplement: Supplementary file 1 [file vaccines-12-00675-s001.zip › vaccines-3019073-Table S1.pdf]

Table S1. Current and previous SARS-CoV-2 variants and their characteristics.

WHO label	Earliest documented samples	Date designated as a variant by WHO	Pango lineage (Nextstrain Clade)	Sublineage	RBD mutations compared to Wuhan (AA #319-541)	Other proteins harboring mutations	Rationale for designation as a variant of interest
Alpha	UK, September 2020	December 18 2020 – March 9 2022	B.1.1.7 (20I)	-	N501Y [201]	N, ORF1ab, ORF8 [202]	Increased transmissibility, immune evasion and disease severity [201,203].
Beta	South Africa, September 2020	December 18 2020 - March 9 2022	B.1.351 (20H)	-	K417N, E484, N501Y [204]	E, N, ORF1ab, ORF3a, ORF8 [202]	Increased transmissibility, immune evasion and disease severity [201,204–206].
Gamma	Brazil November 2020	January 11 2021 – March 9 2022	P.1 (20J)	-	K417T, E484, N501Y [207]	N, ORF1ab, ORF3a, ORF8 [202]	Increased transmissibility, immune evasion and disease severity [201,208,209].
Delta	India, October 2020	May 11 2021 – June 7 2022	B.1.617.2 (21I/21J)	-	L452R, T478K [210]	M, N, ORF1ab, ORF3a, ORF7a [202]	Increased transmissibility, immune evasion and disease severity [211–213].
Omicron	Multiple countries incl. South Africa, November 2021	November 26 2021 – March 2023	B.1.1.529 (21K)	BA.1	G339D, S371L, S373P, S375F, K417N, N440K, G446S, S477N, T478K, E484A, Q493R, Q498R, N501Y, Y505H [202,214–216]	E, M, N, ORF1ab, ORF3a [202]	High transmissibility and immune evasion ability, reduced disease severity [217–219].

WHO label	Earliest documented samples	Date designated as a variant by WHO	Pango lineage (Nextstrain Clade)	Sublineage	RBD mutations compared to Wuhan (AA #319-541)	Other proteins harboring mutations	Rationale for designation as a variant of interest
	South Africa, November 2021	? – March 2023	(21L)	BA.2	G339D, S371L, S373P, S375F, T376A, D405N, R408S, K417N, N440K, S477N, T478K, E484A, Q493R, Q498R, N501Y, Y505H and reversion to Wuhan G446 and G496 [202,215].	E, M, N, ORF1ab, ORF3a, ORF6 [202]	Enhanced transmissibility and immune evasion ability, reduced disease severity [220].
	South Africa, January and February 2022 respectively	? – March 2023	(22A/22B)	BA.4/5	BA.2 variants plus L452R, F486V and reversion to Wuhan R493Q revertant [221]	E, M, N, ORF1ab, ORF3a (+ORF6 for BA4 and ORF7a, ORF8 for BA5 respectively) [202]	Impact on transmissibility and disease severity unclear, increased immune evasion capacity [220].
	February 2022	October 2022	(22E)	BQ.1.1. (descendent of BA.5)	BA.5 variants plus R346T, K444T, N460K	N, ORF1ab, ORF9b [202]	Unknown.
	United States, October 2022	January 2023	(23A)	XBB.1.5	G339H, R346T, L368I, S371F, S373P, S375F, T376A, D405N, R408S, K417N,	E, M, N, ORF1ab, ORF3a, ORF6, ORF8, nsp1 [222]	Enhanced transmissibility, and impact on immunity. Disease severity similar to baseline [179,223–225].

WHO label	Earliest documented samples	Date designated as a variant by WHO	Pango lineage (Nextstrain Clade)	Sublineage	RBD mutations compared to Wuhan (AA #319-541)	Other proteins harboring mutations	Rationale for designation as a variant of interest
					N440K, V445P, G446P, N460K, S447N, T478K, E484A, F490P, F490S, Q498R, N501Y, Y505H [222]		
	Unknown, January 2023	April 2023	(23B)	XBB.1.16	BA.2.10.1 and BA.2.75 plus K478R, F486P [226]	E, M, N, ORF1ab, ORF3a, ORF6, ORF8, nsp1 [222]	No evidence of impact on transmissibility, immunity or disease severity [227].
	Unknown, February 2023	August 2023	(23F)	E.G.5.1	G339H, R346T, L368I, S371F, S373P, S375F, T376A, D405N, R408S, K417N, N440K, V445P, F456L, G446P, N460K, S447N, T478K, E484A, S486P, F490S, Q498R, N501Y, Y505H [228]	?	No evidence of impact on transmissibility, immunity or disease severity [227].
	Unknown, July 2023	November 2023	(23I)	BA.2.86	G339D, S371L, S373P, S375F, T376A,		Some evidence that the variant evades antibodies. No evidence of

WHO label	Earliest documented samples	Date designated as a variant by WHO	Pango lineage (Nextstrain Clade)	Sublineage	RBD mutations compared to Wuhan (AA #319-541)	Other proteins harboring mutations	Rationale for designation as a variant of interest
					D405N, R408S, K417N, N440K, S477N, T478K, E484A, Q493R, Q498R, N501Y, Y505H and reversion to Wuhan G446 and G496 [202,215]		changes in clinical severity [38].
	Unknown, August 2023	February 2024	(not assigned)	JN.1	G339D, S371L, S373P, S375F, T376A, D405N, R408S, K417N, N440K, S477N, T478K, E484A, Q493R, Q498R, N501Y, Y505H and reversion to Wuhan G446 and G496, L5455S [38,202,215]	ORF1a, ORF7b [38]	Some evidence that the variant evades antibodies. No evidence of changes in clinical severity [38].

E, Envelope; M, Membrane; N, Nucleocapsid; N/A, not applicable; ORF, Open Reading Frame; PANGO, Phylogenetic Assignment of Named Global Outbreak; RBD, Receptor Binding Domain; WHO, World Health Organization, Unless otherwise stated all variant information compiled from [38,39,228]. All data correct as at 01/03/2024.
